# Salvage surgery combined with descending aorta resection for lung cancer

**DOI:** 10.1186/s40792-019-0675-9

**Published:** 2019-07-22

**Authors:** Masatoshi Kanayama, Yoshinobu Ichiki, Masataka Mori, Hiroki Matsumiya, Yusuke Nabe, Akihiro Taira, Shinji Shinohara, Taiji Kuwata, Masaru Takenaka, Ayako Hirai, Naoko Imanishi, Kazue Yoneda, Eigo Ikushima, Toru Yasutsune, Yosuke Nishimura, Fumihiro Tanaka

**Affiliations:** 1 Second Department of Surgery, University of Occupational and Environmental Health, 1-1 Iseigaoka, Yahatanishi-ku, Kitakyushu, 807-8555 Japan; 2 Cardiovascular Surgery, University of Occupational and Environmental Health, 1-1 Iseigaoka, Yahatanishi-ku, Kitakyushu, 807-8555 Japan

**Keywords:** Salvage surgery, Descending aorta resection, Lung cancer

## Abstract

**Background:**

Recent retrospective studies have shown that salvage surgery can improve survival with acceptable adverse events, and this procedure has been adapted for lung cancer. However, there are no reports demonstrating the efficacy of salvage surgery combined with aortic resection.

**Case presentation:**

A 73-year-old man had received definitive concurrent chemoradiotherapy (carboplatin/paclitaxel, 70 Gy) for lung cancer originated from the left upper lobe and infiltrating the thoracic aorta (cT4N1M0 stage IIIA). Although the tumor has shrunk significantly (ycT4N0M0 stage IIIA), radiation pneumonitis occurred. Due to the steroid therapy, radiation pneumonitis was relieved; however, re-enlargement of the primary tumor was observed during steroid tapering. Nonetheless, the lymphatic and distant metastases were controlled. Moreover, aortic invasion was localized to the periphery of the third branch, and the tumor was considered to be resectable. Intraoperatively, we observed macroscopic evidence of aortic invasion in the periphery of the third branch; thus, left upper lobectomy combined with descending aorta resection was performed under partial extracorporeal circulation. The patient is currently active without any recurrence 21 months post-surgery.

**Conclusions:**

No clear consensus exists regarding salvage surgery combined with aortic resection for primary lung cancer. However, we believe that this surgery may improve the survival of carefully selected patients.

## Background

Salvage surgery is defined as “surgical resection of locally recurrent or persistent tumor after definitive medical treatment for unresectable cases” [1]. Recent retrospective studies have shown that salvage surgery can improve survival with acceptable adverse events, and this procedure has been adapted for lung cancer [2, 3]. However, there are no reports demonstrating the efficacy of salvage surgery combined with aortic resection. Here, we report a case of successful salvage surgery combined with descending aorta resection for lung cancer.

## Case presentation

**Fig. 1:**
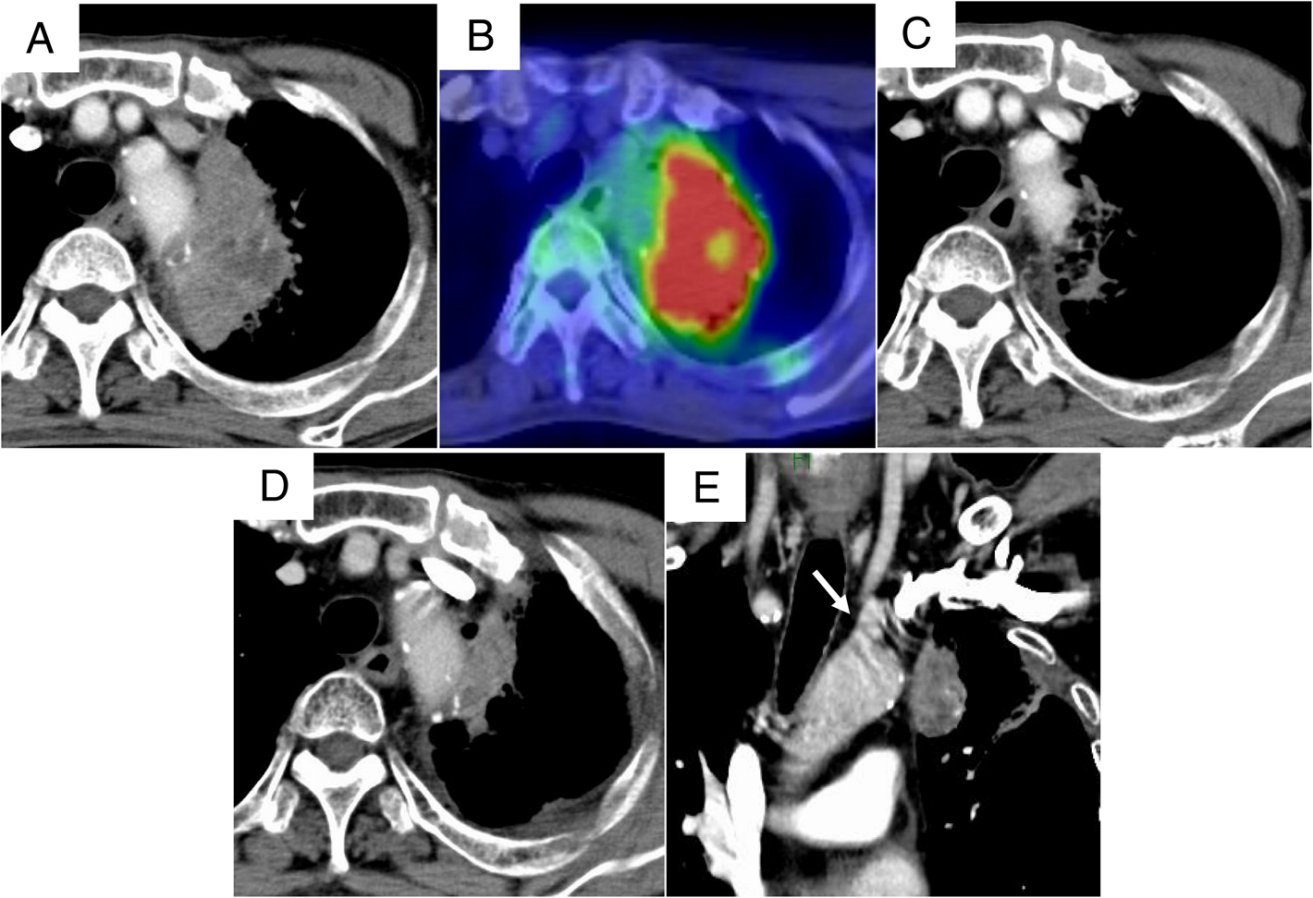
Chest contrast-enhanced computed tomography (CT) and positron emission tomography (PET)/CT findings. **a**, **b** A pulmonary tumor originated from the left upper lobe suspected to have invaded the aorta. **c** Markedly reduced tumor size following definitive chemo-radiotherapy. **d** The re-enlargement of primary tumor during steroid tapering. **e** Aortic invasion localized in the periphery of the left subclavian artery (arrow)

**Fig. 2:**
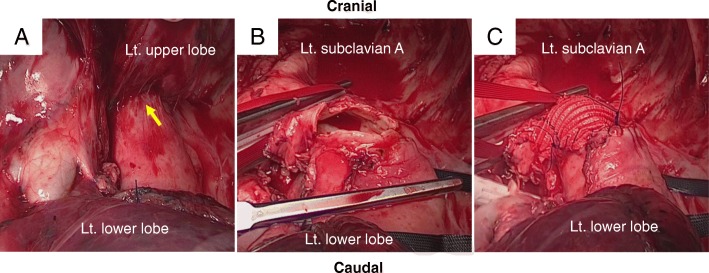
Intraoperative findings. **a** Macroscopic inspection strongly indicating aortic invasion in the periphery of the third branch (arrow). **b** The invasive part of the descending aortic wall is resected under partial extracorporeal circulation. **c** Patch closure of the aortic defect is performed

A 73-year-old man had received definitive concurrent chemoradiotherapy (carboplatin/paclitaxel: 2 courses, 70 Gy) for left upper lobe squamous cell carcinoma suspected to have invaded the aorta (cT4N1M0 stage IIIA; Fig. 1a, b). Although the tumor shrunk significantly (partial response, ycT4N0M0 stage IIIA; Fig. 1c), radiation pneumonitis occurred. Steroid therapy (prednisone, 1.0 mg/kg) was administered, and radiation pneumonitis was relieved. However, re-enlargement of the primary tumor was observed during steroid tapering (Fig. 1d), and the patient was introduced to our department for salvage surgery. The imaging analysis indicated that the lymphatic and distant metastases were controlled, and radiation pneumonitis confined to the left upper lobe was sufficiently controlled by steroid therapy. Although aortic invasion was suspected, it was localized to the periphery of the third branch (Fig. 1e). The patient opted for salvage surgery, which may be curative despite the associated invasiveness and risk, and we planned salvage surgery with continuous steroid administration (prednisone, 0.5 mg/kg, 6 months after definitive chemoradiotherapy). The patient was positioned to keep the upper and lower body in the right lateral and supine positions, respectively. This arrangement allowed cannulation from the inguinal vessels while an anterolateral thoracotomy incision was made through the fifth intercostal space. Macroscopic inspection strongly indicated aortic invasion in the periphery of the third branch (Fig. 2a). The lingular pulmonary artery, superior pulmonary vein, and upper lobe bronchus were dissected from the caudal to the cranial side. Because invasion of the major branches of the main pulmonary artery (A3 and A1 + 2) was suspected, it was resected along with its reconstruction. After performing all procedures (except for dissection at the site of aortic invasion), a cardiovascular surgeon clamped the aorta at the distal site of the left subclavian artery under partial extracorporeal circulation (F-F bypass) and resected an invasive part of the descending aortic wall. Finally, complete resection of the lung cancer that had invaded the aorta was successfully performed. Further, we performed patch closure on the aortic defect (Fig. 2b, c) and covered the site of the aortic patch with an intercostal muscle flap. The duration of the operation and partial extracorporeal circulation were 452 min and 37 min, respectively. The distance between the aortic wall and viable malignant cells was 3 mm, and aortic wall invasion was not recognized as a pathology (EF.2, ypT2aN0M0 stage IB). Although postoperative chylothorax was noted, we could completely control and additional complications were not observed. The patient visited another hospital for rehabilitation 3 months after surgery and has been enjoying daily life without any recurrence for 21 months post-operation.

## Discussion

Local tumor relapse after definitive chemoradiotherapy occurs in up to 35% of non-small cell lung cancer patients and remains the dominant cause of death after initial therapy [4]. Although there is no clear consensus on the effective local treatment modality, previous studies have reported the median progression-free survival (PFS) and overall survival (OS) after salvage surgery to be 12–43.6 months and 22–46 months, respectively [2, 5–7]. The studies also reported that salvage lung resection may be a means of achieving local control and improving survival. However, salvage surgery is generally considered as technically more difficult with potentially higher morbidity than alternative therapies. Therefore, careful patient selection and surgical expertise are both extremely important [8].

Some important points with respect to patient selection for salvage surgery are as follows: (1) adequate pulmonary reserve and good performance status, (2) possibility of complete resection, and (3) controlled lymphatic and distant metastases. In the present case, the patient was able to withstand surgery, and lymph node and distant metastases were controlled. Despite aortic invasion, the tumor was considered to be resectable after careful assessment by cardiovascular surgeons. For lung cancers with aortic invasion, complete resection and no mediastinal lymph node metastases have been identified as predictors of improved survival [9]. Induction chemoradiotherapy has been found to improve resectability and survival [10]. In contrast, only one case of unsuccessful salvage surgery combined with descending aorta resection has been reported [5], and no clear evidence is available supporting the suitability of this operation. However, in our case, controlling the tumor progression (the re-enlargement of the primary tumor) using modalities other than surgery would be difficult. Therefore, we presumed that the prognosis could be improved by complete resection combined with aortic resection.

In cases of combined aortic resection, the surgical strategy varies depending on whether the invaded part is the proximal or distal to the left subclavian artery. If the infiltrated part is distal to the subclavian artery, it can be resected by replacing the distal aortic arch or descending aorta. Conversely, it is necessary to replace the aortic arch if the infiltrated part is proximal to the subclavian artery. Unlike replacing the aortic arch, replacing the distal aortic arch or descending aorta maintains the blood flow to the upper body; hence, only the blood flow to the lower body required external management through partial extracorporeal circulation such as the femoral artery–femoral vein bypass (F-F bypass). Therefore, there is no need for cardiac arrest and extracorporeal circulation of the brain. Extracorporeal circulation time is short, and it can be performed relatively safely without complications such as cerebral infarction. In the present case, the aortic infiltration site was considered to be in the distal to the left subclavian artery. This was the primary reason for performing salvage surgery. In fact, the extracorporeal circulation time was approximately 30 min and complications associated with extracorporeal circulation were not observed.

In this case, we did not perform preoperative thoracic endovascular aortic repair (TEVAR) because the infiltrated part was located close to the left subclavian artery (as seen in a preoperative image), resulting in an insufficient landing zone. However, TEVAR followed by surgery is a less invasive and useful alternative option [11]. Particularly, in cases in which the infiltrated part involves the aorta proximal to the left subclavian artery, aortic arch replacement should be avoided by performing fenestration stent placement [12] or by debranching followed by TEVAR [13]; this is because aortic arch replacement is highly invasive.

No clear consensus exists regarding salvage surgery combined with aortic resection for primary lung cancer. However, we believe that this surgery may improve the survival of carefully selected patients.

## Conclusions

We report the first case of successful salvage surgery combined with descending aorta resection and demonstrate its efficacy for lung cancer.

## Data Availability

The data are not available for public access because of patient privacy concerns but are available from the corresponding author on reasonable request.

## Abbreviations

F-F bypass: Femoral artery–femoral vein bypass
OS: Overall survival
PFS: Progression-free survival
TEVAR: Thoracic endovascular aortic repair
